# Tuberculosis infection risk, preventive therapy care cascade and incidence of tuberculosis disease in healthcare workers at Maputo Central Hospital

**DOI:** 10.1186/s12879-019-3966-7

**Published:** 2019-04-25

**Authors:** Susannah K. Graves, Orvalho Augusto, Sofia Omar Viegas, Philip Lederer, Catarina David, Kristen Lee, Anila Hassane, Anilsa Cossa, Salma Amade, Susete Peleve, Pereira Zindoga, Leguesse Massawo, Francesca J. Torriani, Elizabete A. Nunes

**Affiliations:** 1 Division of Infectious Diseases, Department of Internal Medicine, University of California, San Diego, 9500 Gilman Drive MC 0711, La Jolla, CA 92093-0711 USA; 2 Faculdade de Medicina, Universidade Eduardo Mondlane, Maputo, Mozambique; 3 Instituto Nacional de Saúde, Ministério da Saúde, Maputo, Mozambique; 4 Department of Medicine, Boston University School of Medicine, Boston, MA USA; 5 Department of Internal Medicine, Maputo Central Hospital, Av Agostinho Neto-364, Maputo, Mozambique; 6 UC San Diego Infection Prevention and Clinical Epidemiology and TB Control Units at UC San Diego Health, 200 W Arbor Drive MC 8951, San Diego, California 92103 USA

**Keywords:** Tuberculosis, Latent tuberculosis infection, Interferon-gamma release assay, Healthcare workers, HIV, Isoniazid preventive therapy

## Abstract

**Background:**

Mozambican healthcare workers have high rates of latent and active tuberculosis, but occupational screening for tuberculosis is not routine in this setting. Furthermore, the specificity of tuberculin skin testing in this population compared with interferon gamma release assay testing has not been established.

**Methods:**

This study was conducted among healthcare workers at Maputo Central Hospital, a public teaching quaternary care hospital in Mozambique. With a cross sectional study design, risk factors for tuberculosis were assessed using multivariable logistic regression. The care cascade is reported for participants who were prescribed six months of isoniazid preventive therapy for HIV or highly reactive testing for latent tuberculosis infection. The agreement of interferon-gamma release assay results with positive tuberculin skin testing was calculated.

**Results:**

Of 690 screened healthcare workers, three (0.4%) had active tuberculosis and 426 (61.7%) had latent tuberculosis infection. Less education, age 35–49, longer hospital service, and work in the surgery department were associated with increased likelihood of being tuberculosis infected at baseline (*p* < 0.05). Sex, Bacillus Calmette-Guerin vaccination, HIV, outside tuberculosis contacts, and professional category were not. Three new cases of active tuberculosis developed during the follow-up period, two while on preventive therapy. Among 333 participants offered isoniazid preventive therapy, five stopped due to gastrointestinal side effects and 181 completed treatment. For HIV seropositive individuals, the agreement of interferon gamma release assay positivity with positive tuberculin skin testing was 50% among those with a quantitative skin test result of 5-10 mm, and among those with a skin test result ≥10 mm it was 87.5%. For HIV seronegative individuals, the agreement of interferon gamma release assay positivity with a tuberculin skin test result of 10-14 mm was 63.6%, and for those with a quantitative skin test result ≥15 mm it was 82.2%.

**Conclusions:**

There is a high prevalence of tuberculosis infected healthcare workers at Maputo Central Hospital. The surgery department was most heavily affected, suggesting occupational risk. Isoniazid preventive therapy initiation was high and just over half completed therapy. An interferon gamma release assay was useful to discern LTBI from false positives among those with lower quantitative tuberculin skin test results.

**Electronic supplementary material:**

The online version of this article (10.1186/s12879-019-3966-7) contains supplementary material, which is available to authorized users.

## Background

Tuberculosis disease (TB), with its high morbidity and mortality, depletes human resources in high burden countries [1, 2]. Mozambique has a high TB burden, with 159,000 active cases in 2016. HIV is highly prevalent, affecting 45% of Mozambicans with TB [3].

Worldwide, healthcare workers (HCW) in low and middle income countries have high rates of latent TB infection (LTBI) [4, 5] and recent reviews have made the case for focusing on infection control practices in these settings including routine screening and reporting of TB among workers at health facilities [4, 6].

Mozambican HCW have high rates of active TB and LTBI. A recent study among 209 HCW in Nampula, Mozambique showed an LTBI rate of 34.4% [7] and another among 505 HCW in nearby South Africa demonstrated rates of 62–84% [8]. Although Mozambique adapted its tuberculosis infection prevention and control guidelines to comply with the World Health Organization’s (WHO) 2009 policy [9], these measures are inconsistently implemented in Mozambican health facilities [10]. At Maputo Central Hospital (MCH), occupational TB has been a significant challenge, prompting implementation of tuberculosis infection prevention and control measures, including forming the Nucleus of Tuberculosis Control in 2011 with a mandate to screen and treat workers for TB and LTBI.

Throughout the world, identification of HCWs who may benefit from chemoprophylaxis with isoniazid preventive therapy (IPT) has historically been performed using the tuberculin skin test (TST). More recently, interferon-gamma release assays (IGRAs) are being implemented. Few prior studies in sub-Saharan African HCWs have used IGRA responses to evaluate the burden of LTBI. While occupational risk factors associated with LTBI have been identified [4, 5, 11–14], there has only been one study to date in Mozambican HCW [7].

This study aimed to establish the burden of active TB disease among HCWs at MCH and identify those at highest risk of being TB infected. It further assessed the care cascade of IPT in this setting, as well as agreement of IGRA with positive TST results.

## Methods

### Study design and setting

This is a cross sectional screen for active TB and LTBI with 6 months of follow-up to assess TB incidence and IPT care cascade. MCH is the largest hospital in Mozambique, with 1400 beds and 3679 HCW in 2013. Conditions are crowded and the facility is poorly ventilated. By policy, masks are available to staff and coughing patients but use is neither mandated nor consistent. There is no facility for the isolation of admitted TB suspects. Radiology, pathology and sputum microscopy are available on-site.

### Study population and recruitment

In 2013, all 3679 workers at least 18 years old, working at MCH for at least two years were invited to participate. Participants were recruited through posted flyers and informational sessions. Exclusion criteria were as follows: immune-suppressive therapy with steroids for over four weeks, recent live vaccine, prior active TB, or history of ulceration with TST. Of the 777 healthcare workers who responded, 758 were eligible and provided informed consent. Additional informed consent was obtained for those agreeing to HIV testing.

### Demographics and risk assessment

Demographics and tuberculosis exposure history were collected via interviews in Portuguese by study physicians at enrollment. Age, sex, education level, length of hospital service, department, professional category, Bacillus Calmette-Guerin (BCG) vaccination status and exposure to close contacts with active TB were solicited from each participant.

### Active TB screening

Symptoms of cough greater than two weeks, productive cough, hemoptysis, chest pain, fever, sweats, weight loss or weakness were solicited. All participants, with the exclusion of pregnant women, underwent a chest X-ray which was interpreted by a study radiologist or pulmonologist who was blinded to the symptom questionnaire. Those with any symptoms or suspicious X-ray findings underwent further testing, including sputum collection. Sputum samples were analyzed with fluorescence microscopy using Auramine O staining procedure, and nucleic acid amplification testing (GeneXpert, Cepheid) followed by mycobacterial culture in liquid media (BBL™ MGIT™, Becton, Dickinson and Company) and solid Löwenstein-Jensen media slants (BBL™ Lowenstein-Jensen Medium, Becton, Dickinson and Company). Further workup of computed tomography, bronchoscopy, and/or fine-needle aspiration were performed as indicated by a study pulmonologist.

Active TB cases were defined as positive laboratory testing (smear, culture, or nucleic acid amplification test) in a patient with suggestive symptoms and/or imaging.

### Laboratory testing for HIV and LTBI

All workers agreeing to HIV testing underwent HIV 1/2 immunochromatographic lateral flow antibody testing (Determine™ HIV-1/2 test, Abbott Laboratories). Initially reactive samples underwent confirmatory testing with an immunochromatographic assay (UniGold™ HIV-1/2 test, Trinity Biotech) according to the Mozambican national HIV testing algorithm. Those with confirmed HIV underwent CD4 T-cell count.

Participants in whom active TB was ruled out underwent testing for LTBI using a two-step process. First, a quantitative TST was performed. For those with a TST induration of at least 10 mm (if HIV-) or at least 5 mm (if HIV+), an IGRA (QuantiFERON**®**-TB Gold, Cellestis) was performed when they returned for TST reading at 72 h.

### Latent tuberculosis classification

LTBI classifications are outlined in Table 1. HIV seronegative participants with TST induration of 10–14 mm or quantitative IGRA 0.35–0.99 IU/mL were designated as LTBI “low reactors” along with HIV seropositive participants with TST of 1-4 mm. HIV seropositive participants with TST induration of at least 5 mm, and those with a negative HIV antibody test and a TST of at least 15 mm together with an IGRA result of at least 1.0 were designated LTBI “high reactors.” Those who declined HIV testing but had TST induration of ≥1 mm were classified as LTBI “undetermined reactor status.”

**Table 1:** Latent tuberculosis classification

HIV serostatus	TST induration	TST qualitative	IGRA IU/mL	LTBI Class
Negative HIV Ab				
Negative	0-9 mm	Negative	n/a	No LTBI
Negative	10-14 mm	Positive	Any	Low reactor
Negative	≥15 mm	Positive	0.35–0.99	Low reactor
Negative	≥15 mm	Positive	≥1.00	High reactor
Positive HIV Ab				
Positive	0	Negative	n/a	No LTBI
Positive	1-4 mm	Positive	Any	Low reactor
Positive	≥5 mm	Positive	Any	High reactor
Unknown HIV Ab				
Unknown	0	Negative	n/a	No LTBI
Unknown	1-9 mm	Uninterpretable	Any	Undetermined
Unknown	10-14 mm	Positive	Any	Undetermined
Unknown	≥15 mm	Positive	0.35–0.99	Undetermined
Unknown	≥15 mm	Positive	≥1.00	High reactor

### IPT treatment assignment

After excluding active TB disease, all participants newly diagnosed with HIV were prescribed isoniazid 300 mg daily for six months according to contemporary Mozambican infection control and HIV/TB treatment guidelines [9, 15]. Additionally, those designated as “high reactor” LTBI were offered IPT.

### Statistical analysis

The agreement of IGRA positivity with positive TST results was reported for the baseline screening, stratified by quantitative TST response. Frequencies of participant characteristics were reported at baseline as well as follow up. LTBI at baseline (defined above) was compared for the following risk factors: sex, age category, education level, duration of hospital service (five-year increments), work type, department, exposure to close contacts with TB outside of work, and HIV status. For dichotomous variables, chi-squared analysis was used. For variables with more than two categories univariate logistic regression with Wald chi-squared test statistic to evaluate for overall significance was used. Unadjusted odds ratio (OR), 95% confidence interval (CI) and *p*-value were reported. To compare the adjusted odds of LTBI in various departments, a multivariable logistic regression model was generated using department as the main effect and adjusting for covariates that were found to have *p* < 0.2 in the univariate analysis: age category, education, duration of service, and exposure to TB contacts outside of work. Significant interactions between age category and duration of hospital service as well as age group and department were identified, so a model stratified by age group was also generated (See Additional file 1). ORs and 95% CIs for the main effect as well as two-tailed *p*-values were reported. Given that loss-to-follow-up at 6 months was nearly 50%, baseline characteristics of those who did and did not follow up were compared using a chi-squared test statistic. Active tuberculosis incidence was calculated using the denominator of all who followed up multiplied by the duration of follow-up (6 months). All testing for significance was done using two-tailed alpha-level 0.05. Hosmer-Lemeshow goodness-of-fit testing was performed and tolerances calculated for the model to assess for collinearity. Outliers identified by Pearson Residuals were investigated. Statistical analysis was performed using SAS**®** Studio statistical software, version 3.6.

## Results

### Population characteristics

**Fig. 1:**
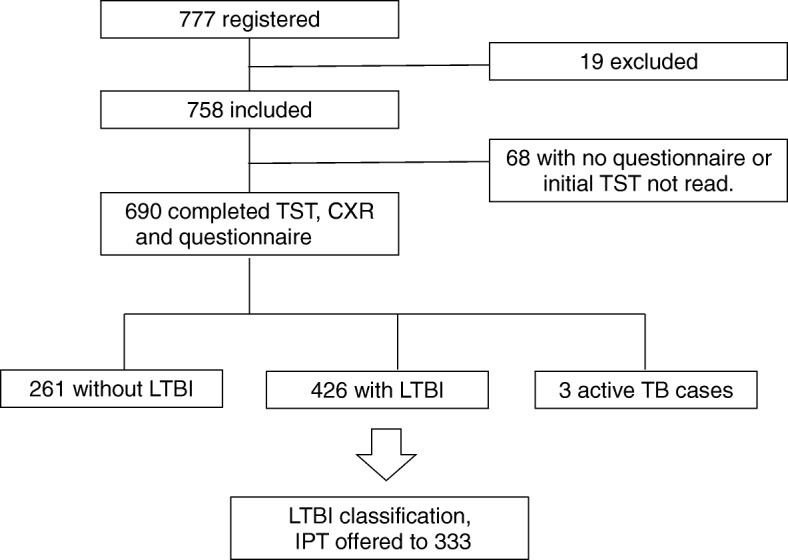
Study participation flow diagram

Of 777 workers screened for participation, 758 met criteria for enrollment in the study, and 690 completed the screening process which included a symptom questionnaire, chest X-ray, and testing for tuberculosis and HIV (Fig. 1).

Baseline characteristics are summarized in Table 2. Women comprised 74.2% of participants. Represented professional categories included auxiliary personnel (49.0%), nurses (17.7%), administrative personnel (14.8%), doctors (6.8%), laboratory (4.1%) and other (7.7%) staff. Of the 645 participants who underwent HIV testing, 78 (12.1%) tested positive, representing 11.3% of the whole sample.

**Table 2:** Baseline characteristics of study participants

Baseline Characteristic	N (%) Total = 690
Sex	
Male	178 (25.8)
Female	512 (74.2)
Age (years)	
Less than 35	227 (32.9)
35–49	303 (43.9)
50 or more	160 (23.2)
Education Level	
Primary or less	169 (24.5)
Secondary	405 (58.7)
College/University	116 (16.8)
Known outside TB contact	
No TB contact	565 (81.9)
TB contact	125 (18.1)
BCG scar	
Absent	54 (7.8)
Present	606 (87.8)
Unsure/missing	30 (4.3)
HIV status	
HIV+	78 (11.3)
HIV-	567 (82.2)
Missing/declined testing	45 (6.5)
Professional category	
Administrative	102 (14.8)
Physician	47 (6.8)
Nurse	122 (17.7)
Laboratory Technician	28 (4.1)
Auxiliary Staff	338 (49.0)
Other	53 (7.7)
Department	
All Non-Clinical	97 (14.1)
Medicine	110 (15.9)
Obstetrics and Gynecology	111 (16.1)
Pediatrics	85 (12.3)
Surgery	107 (15.5)
Clinical Labs and Pathology	61 (8.8)
Emergency and Critical Care	33 (4.8)
Other Clinical Departments	86 (12.5)
Length of Service (years)	
Less than 20	453 (65.7)
20 or more years	237 (34.4)
Baseline Tuberculosis Status	
No LTBI	261 (37.8)
LTBI, low reactor	127 (18.4)
LTBI, high reactor	285 (41.3)
LTBI, undetermined reactor	14 (2.0)
Active TB	3 (0.4)

### Tuberculosis diagnosis

Diagnosis of tuberculosis and LTBI at baseline screening is summarized in Table 2. Three cases of active TB were diagnosed at screening (0.4%) as well as 426 (61.8%) individuals with LTBI, 285 of whom were high reactors (41.3% of total participants). The remaining 261 (37.8%) participants were classified as “no LTBI.” Three additional active TB cases were diagnosed during follow up. The six active TB cases, described in Table 3, were from five different departments.

**Table 3:** Clinical Characteristics of active TB cases

Characteristic	Evaluated in n (Total *n* = 6)	Positive (abnormal) finding
Clinical Type		
Pulmonary	6	4
Lymphadenitis	6	1
Pulmonary + Lymphadenitis	6	1
Baseline Screening		
Symptom Screen	6	1
Chest Xray	6	4
TST	6	4
IGRA	5	4
Mycobacterial Lab Studies		
Acid Fast Stain (sputum or FNA)	3^a^	3
Mycobacterial culture (sputum)	1^b^	1
Nucleic Acid Amplification (sputum)	5	4

^a^Sputum was obtained from the other 3 individuals including induced specimens from 2 individuals, but these were inadequate for evaluation (saliva)

^b^An additional sputum culture was performed but results could not be interpreted due to contamination

### Univariate analysis

The univariate analysis comparing odds of LTBI is shown in Table 4. Age was significantly associated with LTBI, with those 35 to 49 years of age more likely to be positive for LTBI compared with those less than 35 years, OR 1.66 (95% CI 1.16, 2.37). Secondary or higher education was associated with lower odds of LTBI compared with the reference group of primary education or less, ORs of 0.63 (95%CI 0.43, 0.93) and 0.57 (95%CI 0.35, 0.94) respectively. The odds of LTBI increased with service duration with OR 1.12 (95% CI 1.04, 1.21) for each additional 5 years of service. Odds of LTBI were higher in the surgery department with OR 2.43 (95% CI 1.31, 4.49) and were lower in the medicine department with OR 0.53 (95% CI 0.30, 0.92) compared with non-clinical departments. Odds of LTBI did not vary by sex, professional category, BCG vaccination status, outside TB contacts or HIV status.

**Table 4:** Univariate analysis with unadjusted odds ratio of LTBI for specified baseline characteristics

Characteristic	OR (95% CI)	*p*-value
Sex		0.530
Male	ref	
Female	1.13 (0.79, 1.60)	
Age (years)		0.016
Less than 35	ref	
35–49	1.66 (1.16, 2.37)	
50 or more	1.15 (0.76, 1.73)	
Education Level		0.038
Primary or less	ref	
Secondary	0.63 (0.43, 0.93)	
College/University	0.57 (0.35, 0.94)	
BCG scar		0.379
Absent	ref	
Present	0.75 (0.41, 1.36)	
Known outside TB contact		0.125
No TB contact	ref	
TB contact	0.72 (0.49, 1.07)	
HIV status		1.000
HIV-	ref	
HIV+	1.00 (0.61, 1.64)	
Professional category		0.500
Administrative	ref	
Physician	0.62 (0.31, 1.24)	
Nurse	1.03 (0.60, 1.77)	
Laboratory Technician	1.36 (0.56, 3.31)	
Auxiliary Staff	1.15 (0.73, 1.81)	
Other	1.03 (0.52, 2.05)	
Department		< 0.001
All Non-Clinical	ref	
Obstetrics and Gynecology	1.15 (0.66, 2.01)	
Pediatrics	1.06 (0.58, 1.92)	
Surgery	2.43 (1.31, 4.49)	
Medicine	0.53 (0.30, 0.92)	
Clinical Labs and Pathology	1.45 (0.74, 2.86)	
Emergency and Critical Care	1.79 (0.75, 4.27)	
Other Clinical	0.93 (0.52, 1.69)	
Length of Service (5 year increase)	1.12 (1.04, 1.21)	0.002

### Multivariable logistic regression analysis

Summary statistics for the multivariable logistic regression model adjusted for age, education level, TB contact outside of work, service duration and department are shown in Table 5. Longer duration of hospital service was associated with increased odds of LTBI with OR 1.17 (95%CI 1.04, 1.31) for 5-year intervals, as was work in the surgery department with OR 2.31 (95%CI 1.27, 4.44). Age over 50 years was associated with reduced odds of LTBI with OR 0.48 (95% CI 0.26, 0.89), as was work in the medicine department with OR 0.56 (95%CI 0.31, 0.98). A multivariable logistic regression model stratified by age group is described in the supplement [see Additional file 1].

**Table 5:** Odds of LTBI adjusted for age, education, outside TB contact, length of service and department

Characteristic	OR (95% CI)	*p*-value
Age (years)		0.002
less than 35	ref	
35–49	1.15 (0.76, 1.75)	
50 or more	0.48 (0.26, 0.89)	
Education Level		0.139
Primary or less	ref	
Secondary	0.64 (0.41, 0.99)	
College/University	0.70 (0.38, 1.27)	
Known outside TB contact		0.313
No TB contact	ref	
TB contact	0.80 (0.53, 1.23)	
Length of Service (5 year increase)	1.17 (1.04, 1.31)	0.007
Department		0.001
All Non-Clinical	ref	
Obstetrics and Gynecology	1.07 (0.59, 1.91)	
Pediatrics	1.02 (0.55, 1.89)	
Surgery	2.37 (1.27, 4.44)	
Medicine	0.56 (0.31, 0.98)	
Clinical Labs and Pathology	1.57 (0.78, 3.16)	
Emergency and Critical Care	1.82 (0.75, 4.43)	
Other Clinical	0.95 (0.52, 1.74)	

Hosmer-Lemeshow goodness-of-fit test *p*-value = 0.337

### Six-month follow up

Of the 687 participants without active TB disease who completed initial screening, 358 (52.1%) reported for repeat evaluation at the six-month follow up interval. Active TB incidence among those who reported for evaluation at six months was three in 179 person-years, or 1676/100,000 person-years (95% CI 350, 4820). A summary comparing baseline characteristics of those who followed up compared with those who did not is shown in Table 6. Participants who followed up did not differ from those who did not follow up with respect to education level, outside TB contacts, HIV status and professional category. However, males, younger participants, those who worked less than 20 years, and those without LTBI at baseline were less likely to report for follow up.

**Table 6:** Comparison of baseline characteristics between those who did and did not follow up

Baseline Characteristic	No Follow-up N (%) Total = 329	Follow-up N (%) Total = 358	*p*-value
Sex			< 0.001
Male	106 (32.2)	71 (19.8)	
Female	223 (67.8)	287 (80.2)	
Age (years)			0.001
Less than 35	129 (39.2)	96 (26.8)	
35–49	137 (41.6)	165 (46.1)	
50 or more	63 (19.1)	97 (27.1)	
Education Level			0.037
Primary or less	69 (21.0)	99 (27.7)	
Secondary	195 (59.3)	209 (58.4)	
College/University	65 (19.8)	50 (14.0)	
Known outside TB contact			0.636
No TB contact	272 (82.7)	291 (81.3)	
TB contact	57 (17.3)	67 (18.7)	
HIV status			0.243
HIV+	36 (10.9)	40 (11.2)	
HIV-	266 (80.9)	300 (83.8)	
Missing/declined	27 (8.2)	18 (5.0)	
Length of Service (years)			< 0.001
Less than 20	239 (72.6)	211 (58.9)	
20 or more years	90 (27.4)	147 (41.0)	
Professional category			0.300
Administrative	49 (14.9)	53 (14.7)	
Physician	28 (8.5)	19 (5.3)	
Nurse	61 (18.5)	61 (17.0)	
Laboratory Technician	16 (4.9)	12 (3.4)	
Auxiliary Staff	148 (45.0)	188 (52.5)	
Other	27 (8.2)	25 (7.0)	
Department			< 0.001
All Non-Clinical	38 (11.6)	59 (16.5)	
Obstetrics and Gynecology	32 (9.7)	79 (22.1)	
Pediatrics	56 (17.0)	29 (8.1)	
Surgery	48 (14.6)	58 (16.2)	
Medicine	54 (16.4)	55 (15.4)	
Clinical Labs and Pathology	35 (10.6)	25 (7.0)	
Emergency and Critical Care	15 (4.6)	18 (5.0)	
Other Clinical	51 (15.5)	35 (9.8)	
Baseline Tuberculosis Status			< 0.001
No LTBI	156 (47.4)	105 (29.3)	
LTBI, low reactor	58 (17.6)	69 (19.3)	
LTBI, high reactor	106 (32.2)	179 (50.0)	
LTBI, undetermined reactor	9 (2.7)	5 (1.4)	
Active TB	excluded	excluded	

### IPT care cascade

**Fig. 2:**
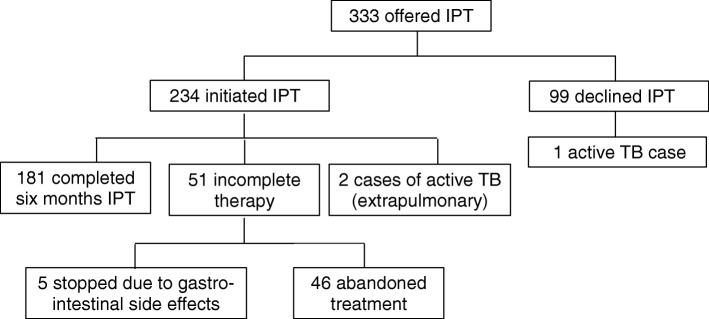
Care cascade of participants who were offered IPT

A flow diagram of the care cascade for the 333 participants offered IPT is shown in Fig. 2. Of 333 participants offered IPT, 234 initiated and 99 declined. Of initiators, 181 completed six months, five stopped due to gastrointestinal side effects, and 46 abandoned treatment. Two cases of drug-sensitive extrapulmonary TB developed during follow-up in participants receiving IPT. One case of active pulmonary TB developed in a participant who declined IPT.

### Operating characteristics of TST and IGRA

The agreement of positive IGRA with positive TST, stratified by quantitative TST response, is shown in Table 7. For HIV seropositive individuals, the agreement of positive IGRAs with positive TST results was 50% among those with TST 5-10 mm, and 87.5% among those with TST ≥ 10 mm. For HIV seronegative individuals, the agreement of TST 10-14 mm with positive IGRA results was 63.6%, and for TST ≥ 15 mm it was 82.2%.

**Table 7:** Agreement of IGRA positivity with TST positive results

	HIV+			HIV-		
TST result (mm)	TST+ *n* = 28	IGRA +	Agreement of IGRA+ with TST+	TST+ *n* = 308	IGRA +	Agreement of IGRA+ with TST+
total	28	23	82.1%	308	243	78.9%
5–9	4	2	50.0%	–	–	–
10–14	8	7	87.5%	55	35	63.6%
≥ 15	16	14	87.5%	253	208	82.2%

## Discussion

Mozambique is among the countries with the highest burden of HIV and TB in the world. At the time of this study in 2013 the national prevalence of HIV was 11.5%. The national incidence of TB was 552/100,000 persons in Mozambique in 2013 [16]. Health workers are at especially risk of TB due both to frequent exposure to patients with infectious TB and because they may also be immunocompromised due to HIV.

In this study we observed 6 cases of tuberculosis, 3 of which were diagnosed during follow-up. The resulting calculated annual incidence of 1676/100,000 persons shows a trend toward higher active TB incidence than the background annual incidence. The incidence of active TB among HCW at MCH is comparable to the median incidence of 1180/100,000 reported in a meta-analysis of active TB in HCW from high-incidence countries [17]. Similarly, in a study conducted in Tete, Mozambique in 2010, 21% of HCW reported prior TB disease with 2.1% diagnosed with active TB disease at screening or during follow up (incidence not specified) [18]. Therefore, our findings support previous work suggesting occupational risk plays a major role in this setting.

One of the limitations of our study is that nearly half of participants did not follow up, including a significantly higher proportion of those without LTBI at baseline. This may have resulted in an overestimation of the incidence of active TB disease.

Much of TB transmission in MCH is likely from unsuspected cases, particularly on services where the chief complaint may be unrelated to TB. In a review of 653 autopsies at MCH, 69 cases of tuberculosis were diagnosed that were unrecognized prior to autopsy, and 44 of these were pulmonary cases [19]. The “Find Actively, Separate and Treat” (FAST) protocol [20] was implemented: patient symptom assessments are done at check-in, with masks and priority given to coughing patients, combined with point-of-care sputum microscopy and nucleic acid amplification testing for TB. Additionally, upper-room germicidal ultraviolet units were installed [21] to address this problem. Of note, the odds of LTBI for HCW in the medicine department were significantly lower, possibly due to infection prevention initiatives focusing on this department.

We note that participants aged 35–49 showed the highest rate of LTBI while those in the oldest age group showed lower rates. This may partly reflect the waning interferon-gamma response in older individuals [22] rather than a true lower rate in this group. In the unadjusted analysis, workers with longer service duration were more likely to have LTBI.

In the present study, uptake among HCWs at MCH was only 70%, with 54% completing treatment even though IPT for LTBI reduces the risk of active TB [23] and is recommended by WHO for newly diagnosed HIV+ individuals in high-TB-burden countries [24]. This care cascade is similar to findings in a recent meta-analysis in which 84.4% of HCW initiated and 50.4% completed therapy [25]. However, it compares favorably with completion rates in low- and middle-income countries in the same study (16.7%) as well as those in a recent study in nearby Swaziland [26]. Newer short-course regimens improve completion and have shown promise in similar high-incidence populations [27–30].

Two participants developed extrapulmonary disease while on IPT, suggesting that these manifestations may be easily missed with symptom screening and chest Xray. While there were no serious adverse events, five participants discontinued therapy due to side effects.

Because the protection from active disease conferred by mass IPT is transient in congregate settings with high reinfection risk [31], studies are needed to further define the role of targeted IPT in high burden settings with ongoing exposure to infectious TB.

Lastly, in contrast to a recent study of HCW in South Africa which demonstrated poor agreement between TST and IGRA [32], a high TST response (≥15 mm in HIV- or ≥ 10 mm in HIV+) had over 80% agreement with IGRA positivity in the present study. This affirms that TST remains a valid test in this resource-limited setting. Conversely, there was poor agreement between low positive TST results and positive IGRA results. Although nontuberculous mycobacteria can cause positive TST results, these infections are much less common than TB among persons living with HIV in Mozambique [33].This suggests that IGRAs could be helpful to discern LTBI from false positive TST among those with low positive TST results in this highly BCG vaccinated population.

## Conclusions

HCW at MCH face high rates of both active TB and LTBI with the highest rates of LTBI in the surgery department. In the study population, TST is useful for diagnosing LTBI for those with higher quantitative responses, and IGRA is useful to discern LTBI from false positives among those with lower quantitative TST. IPT acceptance was high, but achieving high completion rates remains a challenge. New short-course preventive therapy regimens seem promising in this regard. Furthermore, two cases of active extrapulmonary TB developed in individuals on IPT. Future interventions to decrease occupational TB through preventive therapy should focus on better routine screening algorithms to identify both pulmonary and extrapulmonary TB. This is particularly important in populations with high HIV prevalence.

## Additional file

Additional file 1: Multivariable logistic regression model stratified by age. (DOCX 21 kb)

## Abbreviations

BCG: Bacillus Calmette-Guérin
CI: Confidence interval
HCW: Healthcare worker
HCWs: Healthcare workers
HIV: Human immunodeficiency virus
IGRA: interferon-gamma release assay
IPT: Isoniazid preventive therapy
LTBI: Latent tuberculosis infection
MCH: Maputo Central Hospital
OR: Odds ratio
TB: Tuberculosis
TST: Tuberculin skin test
WHO: World Health Organization
