# Supplementary material for: Tuberculosis infection risk, preventive therapy care cascade and incidence of tuberculosis disease in healthcare workers at Maputo Central Hospital
Source: BMC Infect Dis. 2019 Apr 25;19:346. doi: 10.1186/s12879-019-3966-7 (PMC6485058; doi:10.1186/s12879-019-3966-7)
Supplement: Supplementary file 1 — Multivariable logistic regression model stratified by age. (DOCX 21 kb) [file 12879_2019_3966_MOESM1_ESM.docx]

ADDITIONAL FILE 1

Supplementary Results:

*Multivariable logistic regression model stratified by age.*

The adjusted OR for TBI for each risk group among participants stratified by age, are shown in Supplementary Table 1. In the multivariable logistic regression model adjusted for education, outside TB contact, and length of service, participants in the surgery department had higher odds of TBI in the youngest age group with an OR of 8.03 (95%CI 2.19, 29.49), however the magnitude diminished and the association became nonsignificant in the 35-49 age group and disappeared completely in the oldest age group. The association of TBI with length of service was significant only in the middle age group with OR 1.41 (95%CI 1.18, 1.69). No significant associations were present in the oldest age group.

Supplementary Table 1. Multivariable logistic regression model, stratified by age group

| **Characteristic** | **<35 years old (n=227)** | |  | **35 - 49 years old (n=303)** | |  | **≥50 years old (n=160)** | |
| --- | --- | --- | --- | --- | --- | --- | --- | --- |
|  | **OR (95% CI)** | **p-value** |  | **OR (95% CI)** | **p-value** |  | **OR (95% CI)** | **p-value** |
| Education Level |  | 0.450 |  |  | 0.456 |  |  | 0.594 |
| Primary or less | ref |  |  | ref |  |  | ref |  |
| Secondary | 0.46 (0.13, 1.59) |  |  | 0.68 (0.33, 1.39) |  |  | 0.69 (0.33, 1.42) |  |
| College/University | 0.44 (0.11, 1.71) |  |  | 0.93 (0.36, 2.42) |  |  | 0.71 (0.18, 2.80) |  |
|  |  |  |  |  |  |  |  |  |
| Known outside TB contact |  | 0.333 |  |  | 0.092 |  |  | 0.377 |
| No TB contact | ref |  |  | ref |  |  | ref |  |
| TB contact | 0.68 (0.31, 1.49) |  |  | 0.54 (0.27, 1.11) |  |  | 1.43 (0.65, 3.17) |  |
|  |  |  |  |  |  |  |  |  |
| Length of Service (5 year increase) | 1.13 (0.79, 1.62) | 0.491 |  | 1.41 (1.18, 1.69) | <0.001 |  | 0.96 (0.80, 1.16) | 0.691 |
|  |  |  |  |  |  |  |  |  |
| Department |  | 0.014 |  |  | 0.007 |  |  | 0.922 |
| All Non-Clinical | ref |  |  | ref |  |  | ref |  |
| Obstetrics and Gynecology | 1.51 (0.50, 4.54) |  |  | 0.87 (0.34, 2.18) |  |  | 0.79 (0.25, 2.51) |  |
| Pediatrics | 1.34 (0.46, 3.95) |  |  | 1.44 (0.50, 4.11) |  |  | 0.50 (0.14, 1.76) |  |
| Surgery | 8.03 (2.19, 29.49) |  |  | 1.99 (0.77, 5.19) |  |  | 1.04 (0.30, 3.69) |  |
| Medicine | 0.92 (0.34, 2.48) |  |  | 0.52 (0.22, 1.24) |  |  | 0.52 (0.13, 2.08) |  |
| Clinical Labs and Pathology | 1.62 (0.51, 5.15) |  |  | 4.65 (0.91, 23.67) |  |  | 0.77 (0.20, 2.93) |  |
| Emergency and Critical Care | 2.75 (0.73, 10.34) |  |  | 2.77 (0.51, 14.91) |  |  | 0.80 (0.10, 6.16) |  |
| Other Clinical | 3.40 (1.15, 10.02) |  |  | 0.49 (0.19, 1.23) |  |  | 0.55 (0.14, 2.07) |  |
